# Ceruloplasmin in flatland: the relationship between enzyme catalytic activity and surface hydrophilicity

**DOI:** 10.1039/d2ra04159f

**Published:** 2022-09-06

**Authors:** Agata Kowalczyk, Cong Yu, Anna M. Nowicka

**Affiliations:** Faculty of Chemistry, University of Warsaw Pasteura St. 1 Warsaw PL-02-093 Poland akowalczyk@chem.uw.edu.pl; State Key Laboratory of Electroanalytical Chemistry, Changchun Institute of Applied Chemistry, Chinese Academy of Sciences Changchun 130022 China; University of Science and Technology of China Hefei 230026 China

## Abstract

The effective immobilization of the enzyme on the substrate surface plays a key role especially in biocatalysis, medicine or industry. Herein, we showed the influence of substrate hydrophilicity on the activity of the physically immobilized ceruloplasmin. To control the hydrophilicity of the substrate, thiols with various terminal groups were used. We have found that the effectiveness of the catalytic process of multimeric protein is the highest in the situation of application of the highly hydrophilic substrate. In the case of physical adsorption, the orientation of the enzyme is random, however the application of the appropriate modifying layer enforces the desired enzyme orientation. The quartz crystal microbalance with dissipation (QCM-D) results showed that the crucial parameter for the highest and most durable catalytic activity of the enzyme is the orientation, not the amount of the physically adsorbed enzyme.

## Abstract 2

Surface hydrophilicity – the way to control the activity of the immobilized enzyme.

## Graphical abstract

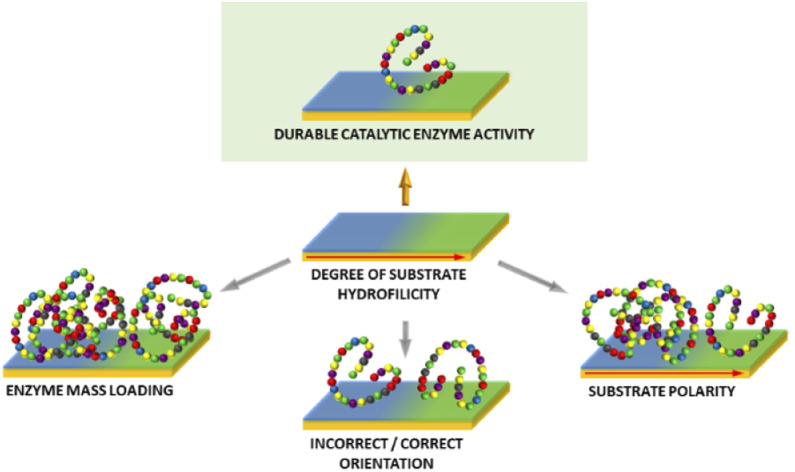

## 1. Introduction

Biotechnological processes using effective catalysts have an important role in various processes and reactions.^1–3^ Most of them use natural catalysts – enzymes, and proteins showing catalytic activity. The numerous advantages of using enzymes as catalysts for chemical reactions have made them applicable in many fields of biotechnology, and biomedicine.^4,5^ However, they can also cause many problems, most of which involve loss of the enzyme activity related to the instability of the structure of the given proteins.^6,7^ The enzymes are sensitive to physicochemical conditions such as pH and temperature, which limits their scope of application. A particularly important and, unfortunately, poorly studied in this respect, group of proteins are soft proteins (also called multimeric/domain proteins) whose domain structure has individual specific properties, such as hydrophilic/hydrophobic, polar/non-polar, or charged/uncharged.^8,9^ Their importance is related to the low conformational stability and the resulting structural changes of the protein taking place during the adsorption process. Moreover, the appropriate orientation of the immobilized enzyme is crucial in achieving the set goal and allows the use of its full potential.^9–13^ It has been proven that the enzyme immobilized on the surface is more stable, making it more resistant to changes in the physical and chemical conditions, which allows its repeated use, and thus in consequence a significant reduction in research costs.^14,15^ The problem of effective immobilization of enzymes with keeping their natural properties on surfaces with different properties is crucial in medicine, biology, and pharmaceutics as well as in food technology, nanobiophotonics and nanobioelectronics.^16–23^ The complexity of this is manifested in terms of permanent and stable immobilization of a certain amount of the enzyme, in the maintenance of their natural properties, and most importantly biological activity. Therefore a given enzyme catalyzes only a few of the many possible reactions for a given substrate. The efficiency of the catalytic process depends directly on the structure of an enzyme, its capacity to transform a specific chemical functionality and its regio- and stereoselectivity.^24^ The availability of the enzyme active site for the reaction substrate is the key factor. Due to the low internal stability of the soft proteins as a result of their contact with the substrate, the conformation may change or the achieved enzyme orientation during the immobilization process will be unfavorable for its action.^25–27^ Both the appropriate orientation of the immobilized enzyme molecules *versus* the surface and their conformation guarantee the preservation of their natural functions, and in the case of electroactive proteins these parameters determine the electron transport mechanism between the immobilized molecule and the electrode surface.^28,29^ Structural changes of enzymes occurring during the adsorption process on the substrate, on the one hand, can lead to the loss of their biological activity and protein denaturation, and on the other hand, to a completely opposite effect, *i.e.* increased biological activity.^30,31^ Taking into account that a great number of important issues involve protein immobilization, a comprehensive analysis of the dependence of multimeric protein activity in the function of the protein–protein and the protein–surface interactions is crucially required. In this context, we focused on the important problem of catalytic activity changes of the multimeric protein, as a result of immobilization process on the substrates differ in the hydrophilicity on the example of ceruloplasmin (Cp). Ceruloplasmin is a domain-structured protein that belongs to the group of soft proteins, that more demanding group of proteins susceptible to structural changes caused by contact with the surface. Such changes are extremely important because the Cp is a metalloprotein whose activity is associated with the redox processes of the active metallic center deeply embedded in the protein shell. Therefore, as a result of structural changes, the active center may become more accessible to the redox process, which would improve the Cp activity. The domain-structured protein molecules immobilized on the hydrophilic surfaces can change their orientation as far as the packing density of the layer allows it so that their hydrophilic domains will orient toward the substrate surface. In contrast, in the case of the enzyme anchored on the hydrophobic surfaces, the hydrophobic domains are involved in the process.^32–34^ The same scheme of behavior takes place when proteins containing differently charged domains adsorb on the charged surface. Adsorption rates are high when protein and surface bear opposite charges, then electrostatic attractions accelerate the migration towards the surface, and the protein orientates itself towards the surface with the domain countercharged to the surface. Neither ceruloplasmin nor any other soft protein has been studied in this respect so far.

In this work, we prove that the hydrophilicity of the substrate is crucial in preserving the enzyme activity, due to the fact that the substrate surface properties influence the arrangement and orientation of the immobilized soft protein (ceruloplasmin). By the application of quartz crystal microbalance and chronoamperometry we proved that the orientation of the immobilized ceruloplasmin, not its amount, is crucial in catalytic activity. Moreover, the experiments using surface plasmon resonance enable the estimation of the affinity constants of the protein–surface interactions in the function of surface hydrophilicity.

## 2. Experimental

### 2.1. Materials and methods

KH_2_PO_4_ (POCH, Poland), K_2_HPO_4_ (POCH, Poland), K_2_SO_4_ (POCH, Poland), 1-octanethiol (HS–C_8_H_17_; Sigma Aldrich), 8-mercapto-1-octanol (HS–C_8_H_16_–OH; Sigma Aldrich), 8-amino-1-octanethiol (HS–C_8_H_16_–NH_2_; Sigma Aldrich), 8-mercaptooctanoic acid (HS–C_7_H_14_–COOH; Sigma Aldrich) and human ceruloplasmin (Cp; Sigma Aldrich) were of the highest purity available. Sodium hydroxide, potassium dihydrogen phosphate, dibasic potassium phosphate, potassium sulphate and potassium ferrocyanide were purchased from Avantor Performance Materials Poland. All solutions were prepared on the basis of ultrapure deionized water with conductivity equal 0.060 μS cm^−1^ (Hydrolab). Due to the fact that chloride ions deactivate the oxidase activity of ceruloplasmin the catalytic measurements (chronoamperometry) were performed in 0.1 M phosphate buffer (PB) of pH 7.0 with addition of 0.15 M K_2_SO_4_.^35^

### 2.2. Applied techniques

The surface plasmon resonance (SPR) experiments were carried out with the use of Biacore X100 from Cytiva. The measurements were made on sensor chip Au (Cytiva) with a flow rate of 1 μL min^−1^. Before the experiments the gold sensor chip was cleaned according to the TL1 procedure. The mixture of ultrapure water, 25% ammonia and 30% hydrogen peroxide in the volume ratio 5 : 1 : 1 was heated to temperature 75 °C, after reaching the set temperature the gold sensor was immersed in this mixture for 5 minutes. Next, the surface of the Au-chip was rinsed with ultrapure water and then with 99.8% ethanol and dried with argon. The self-assembled thiol monolayers were first formed at the gold surface form 1 mM aqueous solutions for 1 h (contact time 3600 s). Next, the system was rinsed with ultrapure water and then the water was changed to 0.01 M PB buffer with the addition of 0.15 M K_2_SO_4_. After that the ceruloplasmin at the appropriate concentration from the range 0.01–1000 pM was injected onto the gold chip modified with the appropriate thiol layer (HS–C_8_H_17_, HS–C_8_H_16_–OH, HS–C_8_H_16_–NH_2_, HS–C_7_H_14_–COOH); association time 60 s and dissociation time 120 s.

The quartz crystal microbalance with dissipation (QCM-D) studies were performed using QCM E4 instrument (Q-Sense from Biolin Scientific) equipped with 4.95 MHz quartz crystals coated with gold (QCM-D crystals, type QSX 301 Gold). The cleaning and modification procedures of those crystals were as for the SPR chip.

Polarization Modulation Infrared Reflection Absorption Spectroscopy (PMIRRAS) experiments were performed on the Thermo Nicolet 8700 (Nicolet, Madison, WI) spectrometer equipped with a custom-made external table-top optical mount, an MCT-A detector cooled with liquid nitrogen (Nicolet), a photoelastic modulator, PEM (PM-90 with II/Zs50 ZnSe 50 kHz optical head, Hinds Instrument, Hillsboro, OR), and a synchronous sampling demodulator, SSD, (GWC Instruments, Madison, WI). The angle of incidence was set at 80°, which gives the maxim of mean square electric field strength for the air/gold interface. Typically 1000 scans were performed and the resolution was set at 4 cm^−1^.

**Scheme 1:**
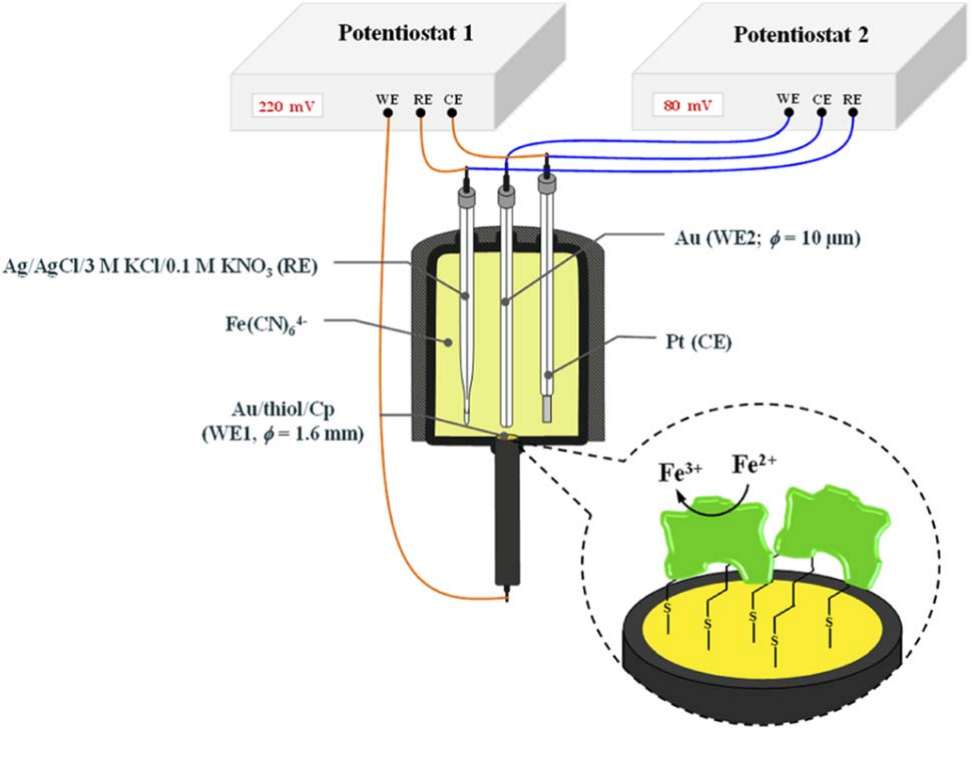
Scheme of the system for chronoamperometric measurements.

The chronoamperometry (ChA) experiments were carried out in a two potentiostat systems using PGSTAT 12 potentiostats (Metrohm-Autolab). The first potentiostat (“potentiostat 1”) was used to maintain the copper active center of ceruloplasmin at the +II oxidation state, and the second one (“potentiostat 2”) was used to monitor the current generated during the catalytic reduction of iron(iii) ions by ceruloplasmin. Both, “potentiostat 1” and “potentiostat 2” are a three-electrode systems consisted of working electrode (in the case of “potentiostat 1” system gold disc electrode (WE1; *ϕ* = 1.6 mm, BASi Instruments), in the case of “potentiostat 2” system gold microelectrode (WE2; *ϕ* = 10 μm, BASi Instruments)), reference electrode (Ag/AgCl/3 M KCl/0.1 M KNO_3_) and counter electrode (platinum plate), according to the Scheme 1.

The reference electrode with a double junction was used to eliminate leakage of chloride ions to the buffer solution and in consequence prevent the ceruloplasmin from the deactivation process. The surface of working electrodes was cleaned in two steps; (i) mechanically by polishing on a wet pad with the addition of 1.0 μm Al_2_O_3_ powder, and (ii) electrochemically by cycling in 0.1 M H_2_SO_4_ aqueous solution in a potential range from −0.3 to 1.5 and back to −0.3 V (*vs.* Ag/AgCl/3 M KCl/0.1 M KNO_3_) with a scan rate 0.05 V s^−1^, until a stable voltammogram, typical for a bare gold electrode, was obtained.

Contact angle measurements were performed using a Hetta Lite Optical Tensiometer, model TL100 for the gold surface modified with appropriate thiol monolayer.

### 2.3. Surface modification

After the cleaning procedure, the surface of gold substrates used in ChA experiments was modified with a thiol layer with different terminal groups (–CH_3_, –OH, –NH_2_, –COOH) formed from 1 mM aqueous solution by chemisorption process for 12 h. Then, the electrode surface was rinsed with a stream of deionized water to remove non-specifically bound thiol chains and dried with a very gentle stream of inert gas. Next, a 6 μL-droplet of 1 μg mL^−1^ ceruloplasmin solution in 0.1 M phosphate buffer with 150 mM K_2_SO_4_, pH 7.0 was placed on the thiol layers and left for 5 h under the cover to prevent the drop from drying out. Such prepared substrate was ready to use.

## 3. Results and discussion

### 3.1. Contact angle analysis

**Fig. 1:**
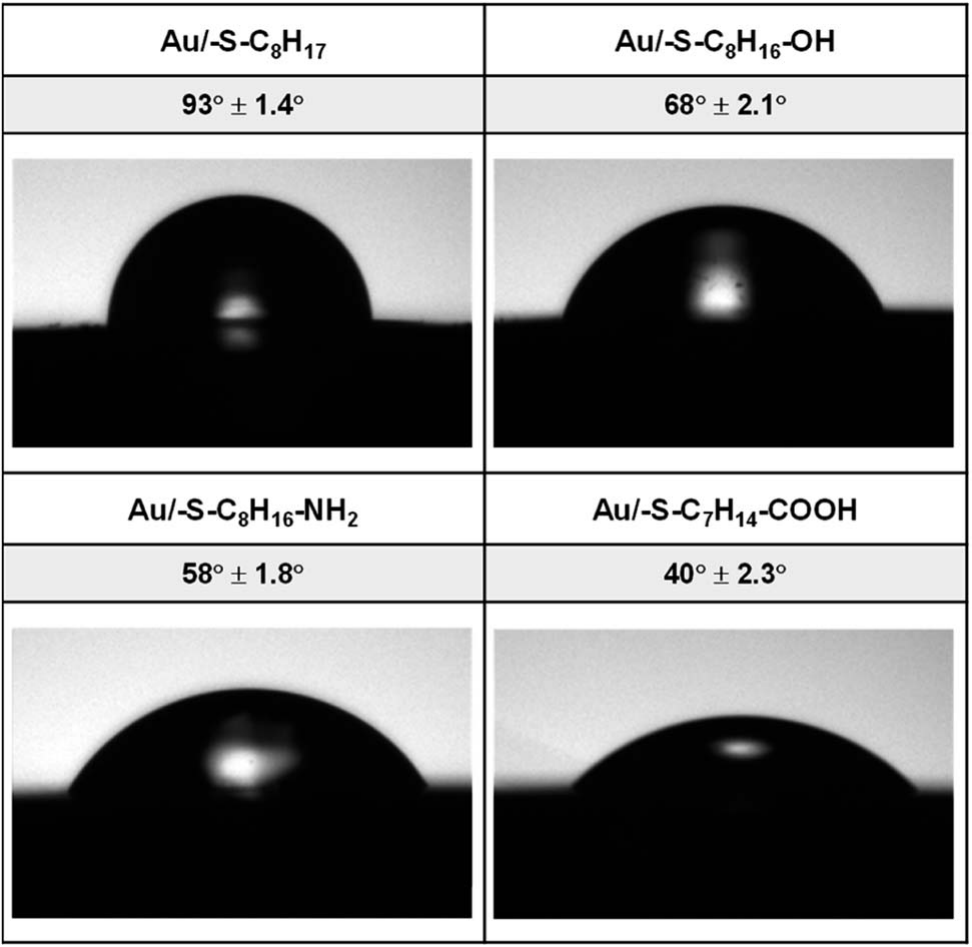
Water contact angles of thiol layers on gold.

The hydrophilicity of the surface depends on the material itself as well as on the type of the layer modifying the surface material. In order to differentiate the hydrophilicity of the gold surface, the thiols with different terminal groups were used. The shape of a water drop and the values of the contact angles obtained for Au/–S–C_7_H_14_–R (*R* = –CH_3_, –CH_2_OH, –CH_2_NH_2_, –COOH) are shown in Fig. 1.

On the basis of the obtained values of the contact angle, it can be concluded that the replacement of the terminal CH_3_ group in the alkanethiol chain with hydrophilic functional groups resulted in a decrease in the value of the contact angle, and thus an increase in the hydrophilic properties of the obtained layers follows the order –OH < –NH_2_ < –COOH.

### 3.2. SPR analysis

**Fig. 2:**
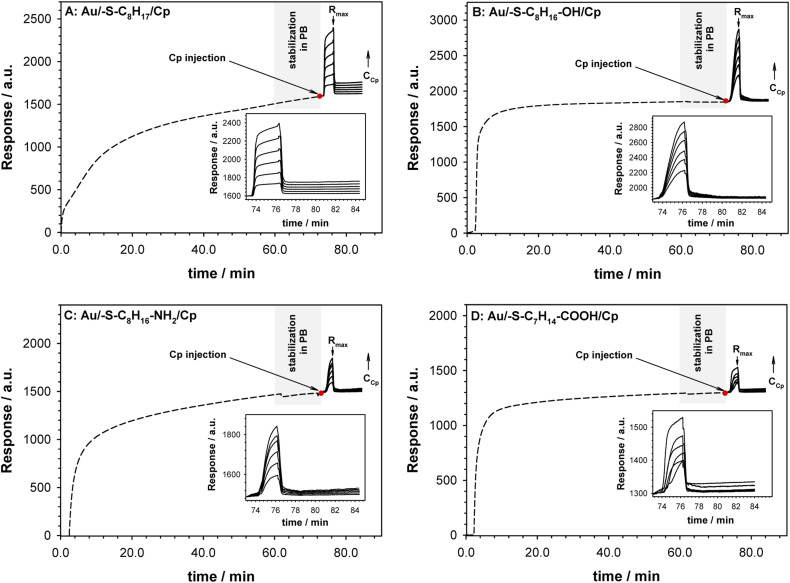
Sensorgrams recorded for Cp interaction (solid lines) with –S–C_8_H_17_ (A), –S–C_8_H_16_–OH (B), –S–C_8_H_16_–NH_2_ (C) and –S–C_8_H_14_–COOH (D) layers (dashed lines). Inset: enlarged selected part of sensorgrams. Experimental conditions: 0.1 M PB (pH 7.0) with addition of 0.15 M K_2_SO_4_, *C*_Cp_ = 0.01–1000 pM, *C*_thiol_ = 1.0 mM, *t*_thiol chemisorption_ = 1 h. Grey rectangle describes the step of stabilization of the system in PB buffer. Red dot mark the moment of Cp injection.

**Fig. 3:**
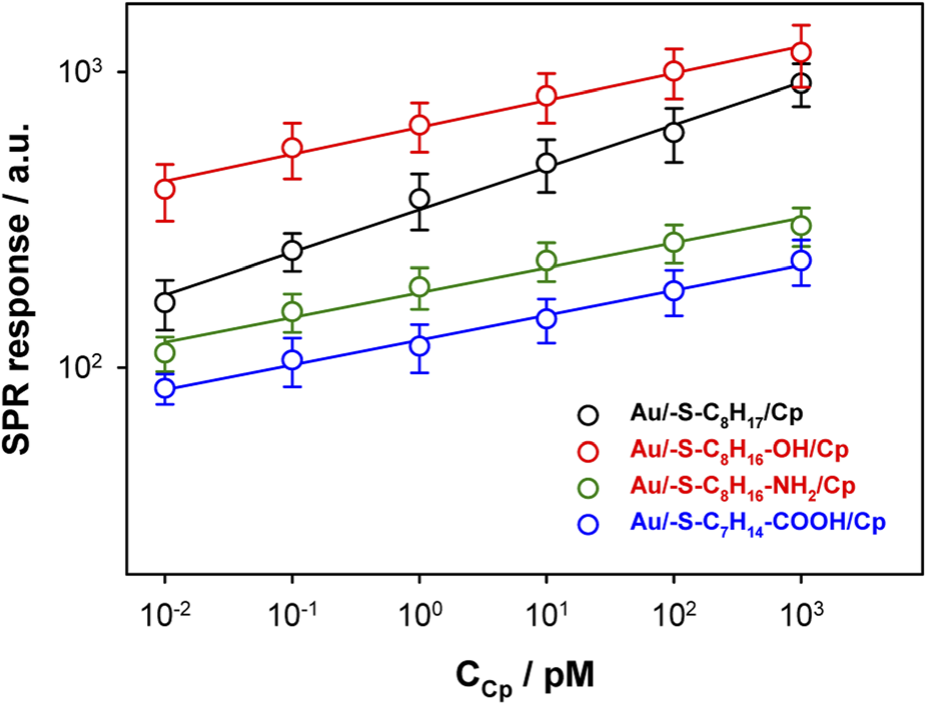
Dependences of SPR responses (based on *R*_max_ values) *versus* ceruloplasmin concentration.

The surface plasmon resonance was applied to determine the influence of the hydrophilicity degree on the binding affinity of the multimeric protein to the substrate surface. SPR analysis of ceruloplasmin interactions with surfaces that differ in degree of hydrophilicity was performed at room temperature in 0.01 M PBS as a running buffer. The changes in the SPR response after injection of Cp, in various concentrations, onto the sensor chip surface are presented in Fig. 2. For all applied thiol-modified substrates the association curves did not exhibit a plateau, which suggests the existence of not only the protein–thiol layer interactions but also protein–protein interactions. The adsorption model that describes the multilayer formation process is the Freundlich-type adsorption isotherm, which is expressed by the equation:1
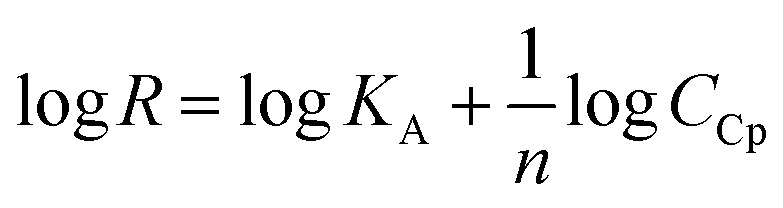
where *R* is a SPR response, *K*_A_ is the equilibrium association constant, *n* represents the heterogeneity of the layer and *C*_Cp_ is the concentration of ceruloplasmin. From the *y*-intercept of the dependences log *R* = f(log*C*_Cp_), presented in Fig. 3, the value of association constants was determined, in turn, the equilibrium dissociation constant (*K*_D_), which is the reciprocal of *K*_A_, was obtained from eqn (2).2
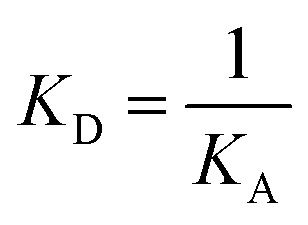


The regression equations describing the linear responses of log*R* = f(log*C*_Cp_), as well as the values of *n*, *K*_A_ and *K*_D_ are presented in Table 1A. The determined data clearly showed that with increasing hydrophilicity degree of the surface the value of affinity constant decreased contrary to the heterogeneity of the Cp layer. The high value of the heterogeneity parameter can be explained by the formation of multilayers as well as the varied orientation of Cp molecules.

**Table 1:** SPR and QCM data concerning the immobilization process of ceruloplasmin onto the thiol layers with various terminal groups

Parameter	Au/–R			
	Au/–S–C_8_H_17_	Au/–S–C_8_H_16_–OH	Au/–S–C_8_H_16_–NH_2_	Au/–S–C_7_H_14_–COOH
**A: SPR data describing adsorption process of Cp on Au/–R layers (*n* = 3)**				
Regression equations	log *R* = 0.14 log *C*_Cp_ + 2.53	log *R* = 0.10 log *C*_Cp_ + 2.81	log *R* = 0.085 log *C*_Cp_ + 2.25	log *R* = 0.082 log *C*_Cp_ + 2.09
*R* ^2^	0.955	0.930	0.966	0.990
*n*	7.1	10.1	11.9	12.2
*K*_A_/[M^−1^]	3.38 × 10^2^	6.46 × 10^2^	1.78 × 10^2^	1.23 × 10^2^
*K*_D_/[M]	2.95 × 10^−3^	1.55 × 10^−3^	5.62 × 10^−3^	8.13 × 10^−3^
*Γ*_SPR_/[pmol cm^−2^]	0.69	0.88	0.23	0.17

**B: QCM-D data describing adsorption process of Cp on Au/–R layers (*n* = 3)**				
Regression equations	Δ*D* = −0.051 Δ*f* − 0.066	Δ*D* = −0.058 Δ*f* − 0.31	Δ*D* = −0.063 Δ*f* − 0.034	Δ*D* = −0.094 Δ*f* − 0.78
*R* ^2^	0.992	0.987	0.997	0.998
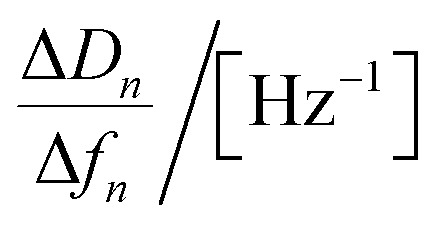	35.0 × 10^−9^	53.9 × 10^−9^	70.7 × 10^−9^	83.5 × 10^−9^
*Γ*_QCM_/[pmol cm^−2^]	7.33	6.32	5.55	6.12
a *Γ*_H_2_O_/[pmol cm^−2^]	6.64	5.44	5.32	5.95

a *Γ*_H_2_O_ = *Γ*_QCM_ − *Γ*_SPR_.

### 3.3. QCM-D and PMIRRAS analysis

**Fig. 4:**
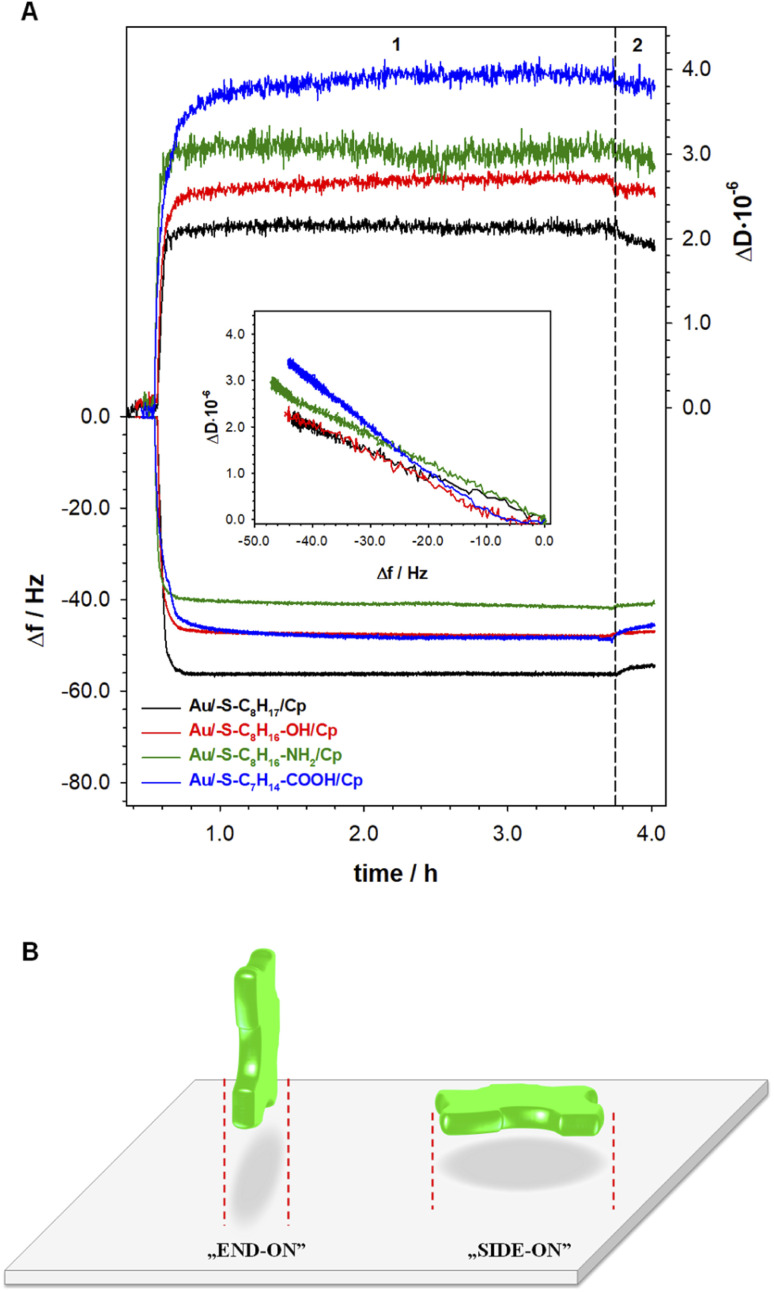
(A) Typical QCM-D spectra of the shifts in frequency (Δ*f*) and dissipation factor (Δ*D*) during formation of Cp layers (step 1) on modified with thiols containing different terminal groups gold electrode surface. Inset: Δ*D versus* Δ*f* plots of Cp layers. Experimental conditions: *C*_Cp_ = 0.20 mg mL^−1^; 0.1 M PB (pH 7.0) with addition of 0.15 M K_2_SO_4_; wash out buffer: 0.1 M PB (pH 7.0) with addition of 0.15 M K_2_SO_4_ (step 2). (B) Possible orientations of Cp at substrate surface.

The information about the arrangement of enzyme molecules in the layer in the function of the hydrophilicity degree of the substrate provides the measurements with using quartz crystal microbalance with dissipation. Typical changes of frequency (Δ*f*) and dissipation (Δ*D*) shifts recorded during ceruloplasmin anchoring to the alkanethiol layer modified QCM-D crystal are shown in Fig. 4. The process of the formation of the self-assembled thiol monolayers on the QCM-D crystals was performed outside the flow chamber. Next, the as-modified QCM-D crystals were placed in the QCM-D chamber. After the frequency stabilization, the ceruloplasmin solution in 0.1 M PB (pH 7.0; with the addition of 0.15 M K_2_SO_4_) was added to the chamber. The addition of ceruloplasmin caused a significant decrease in the frequency and an increase in the dissipation factor related to the formation of the Cp layer. The frequency drop varied for the used thiol layers and was directly associated with the number of attached ceruloplasmin molecules. Whereas, the number of anchored Cp molecules is strictly related to their arrangement in the layer depending on the hydrophobicity of the substrate.

It is generally known, that extent of protein adsorption increases with increasing surface hydrophobicity.^36–40^ It is due to the fact that the hydrophobic surfaces destabilize the protein structure and in consequence facilitate protein conformational rearrangement, which is reflected in stronger protein–protein and protein–surface interaction.^41^ The obtained QCM-D results are in very good agreement with this knowledge. In the case of Cp adsorption onto 1-octanethiol layer (the most hydrophobic of the tested thiol layers) the biggest decrease in Δ*f* – corresponding to the highest surface concentration (7.33 pmol⋅ cm^−2^) was observed (black line in Fig. 4A). Moreover, the changes in dissipation factor recorded during the formation of the Cp layer on the surface of the 1-octanethiol were the smallest, which proves the compactness of the Cp layer. For the remaining thiol layers, the formation of a ceruloplasmin layer was much more complex. Ceruloplasmin is a multimeric protein and depending on the local composition of amino acid can be divided into domains differing in charge, hydrophilicity/hydrophobicity.^8,9,42^

Such feature cause that depending on the surface properties protein predominantly expose domains with properties that fit to surface properties. Therefore, the functional groups of thiol layers determine the orientation/arrangement of Cp molecules in the layer, which is very well illustrated by the dependences of Δ*D versus* Δ*f* in the inset of Fig. 4A. The dependences Δ*D* = f(Δ*f*) obtained for the Cp layers formed at 1-octanethiol, 8-mercapto-1-octanol, and 8-amino-1-octanethiol layers was very similar (Table 1B). Despite negligible differences in the slopes of the curves, the hydrophilicity of the surface, on which the enzyme was immobilized, beard in on the value surface concentration of Cp. As the hydrophilicity of the surface increased, the amount of Cp molecules bound to the surface decreased. The organization of the enzyme layer in the case of the most hydrophilic surface (Au/–S–C_7_H_14_–COOH) was different as evidenced by the value of the slope of the dependence (Δ*D* = f(Δ*f*)) and the value of the ceruloplasmin surface concentration.

Human ceruloplasmin is a multimeric protein containing 6 domains, with dimensions 26.8 × 26.8 × 12.9 nm (*x*, *y*, *z*).^43^ Assuming an elliptical shape of this enzyme we can distinguish two orientations on the surface: side-on and end-on, which are presented in the Fig. 4B. Using the formula for the ellipse area (*A*_theoret. Cp_ = π·*a*·*b*) the theoretical surface area occupied by one, not hydrated molecule of Cp for each orientation is equal to 563.8 (*x*, *y*) and 271.4 nm^2^ (*y*, *z* and *x*, *z*). Taking into account the Cp molecular weight of ∼132 kDa (*M*_W_ = 131 975.98 g mol^−1^), the maximal theoretical surface concentration corresponding to the formation of a 2D ceruloplasmin monolayer for each orientation is equal to 0.294 (side-on) and 0.612 pmol cm^−2^ (end-on). The estimated theoretical *Γ*_max_ (so-called dry mass) value for side-on orientation is in good agreement with the experimental data obtained for thiol layers with –NH_2_ and –COOH terminal groups (see SPR data in Table 1A). For the other two layers (with –CH_3_ and –OH terminal groups) the protein orientation in the layer is end-on. It is known, that proteins adsorb more extensively at hydrophobic than hydrophilic surfaces,^38,39,44,45^ that is why the highest protein surface concentration was obtained at 1-octanethiol layer (see Table 1A). In turn, the hydrophilicity of the surface causes protein–surface interactions, in which electrostatic interactions play a significant role, and are as important as protein–protein interactions.

**Fig. 5:**
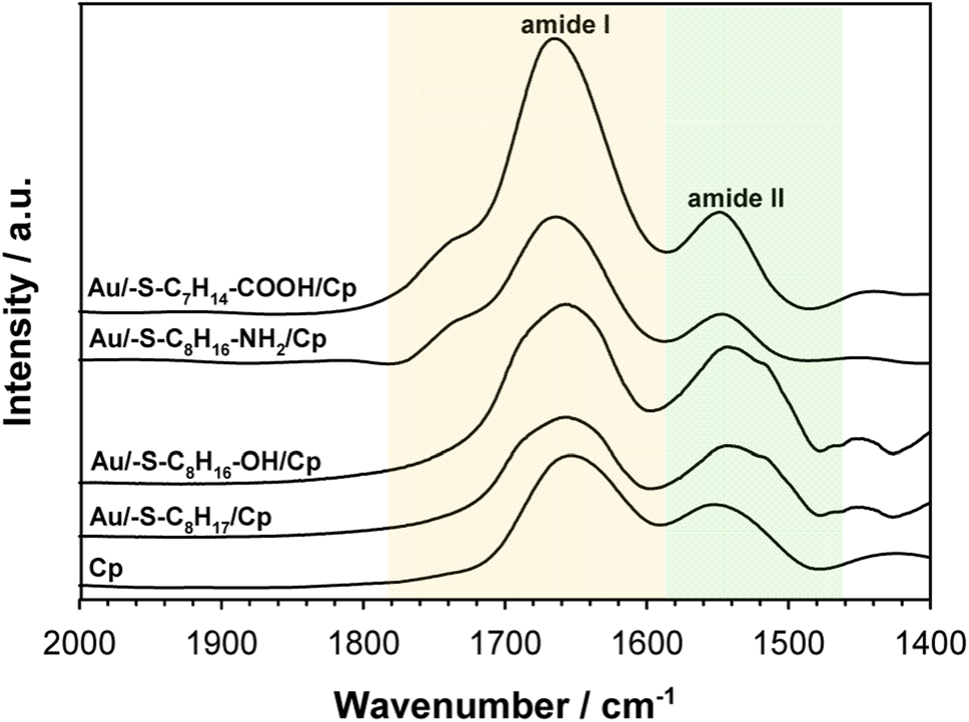
PMIRRAS spectra of ceruloplasmin immobilized on thiol layer with various terminal groups and FTIR spectrum of Cp in KBr. The spectra were recorded in the region of amide I and amide II bands.

To get the information about the influence of the substrate surface hydrophilicity on the enzyme orientation the PMIRRAS experiments were performed. The intensity of the bands in the PMIRRAS technique strongly depends on the orientation of the molecules, more precisely their dipole moments, with respect to the substrate.^46^ The molecules oriented perpendicular to the substrate surface are characterized by the highest signal intensity. The absence of this band or its very weak intensity did not mean the absence of the molecules on the substrate, but only their very different orientation towards the substrate. Fig. 5 shows the Cp spectra recorded after its immobilization on the gold surface modified with appropriate thiol layer and the spectrum of the native enzyme recorded in KBr pellet. The FTIR spectrum of each protein exhibits many characteristic bands called “amide bands”.^47–49^ However, the most useful for the analysis are the bands in the regions: 1600–1700 cm^−1^ (amide I) and 1480–1575 cm^−1^ (amide II). The amide I band is related with the C=O stretching vibration of the amide groups whilst the amide II band corresponds to the N–H bending (40–60%) and C–N stretching (18–40%) vibrations. In studied cases no changes in the position of these two bands was observed, but significant changes in the intensity ratio of the amide I to amide II bands. This ratio increases with the hydrophilicity degree of the substrate surface, it suggest that β sheets in the Cp structure are tilted with respect to the substrate surface. The change in the position of the β sheets is due to a change in the orientation of the enzyme molecules.^50–52^ In consequence the distance between the electrode surface and the active site of Cp also change what should be reflected in the efficiency of the catalytic process.

### 3.4. Chronoamperometric analysis

The catalytic activity of ceruloplasmin, like that of other enzymes, depends on the availability of its active center for the reaction substrate. Unfortunately, the active centers usually are deeply embedded in the protein shell and access to them is difficult. That is why the changes in orientation as well as structural changes of the protein taking place during its immobilization on the surface are so important. The activity of ceruloplasmin is directly connected with the presence of 6 Cu sites in its structure: three of them are located in the T2/T3 cluster and three others formed the three T1-binding sites (T1 Remote, T1CysHis and T1PR).^53^ The T2/T3 cluster is responsible for the electron exchange with the catalytic substrate *e.g.* Fe(ii), whilst the T1-binding sites are responsible for the oxidase activity of Cp.^53–55^

**Scheme 2:**
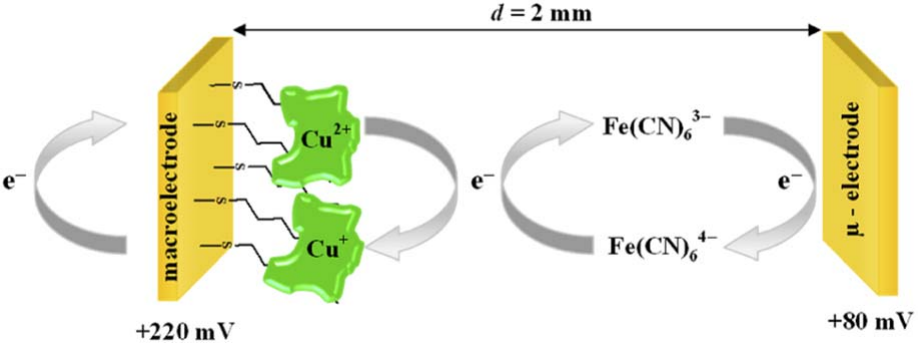
Scheme of the catalysis process.

**Fig. 6:**
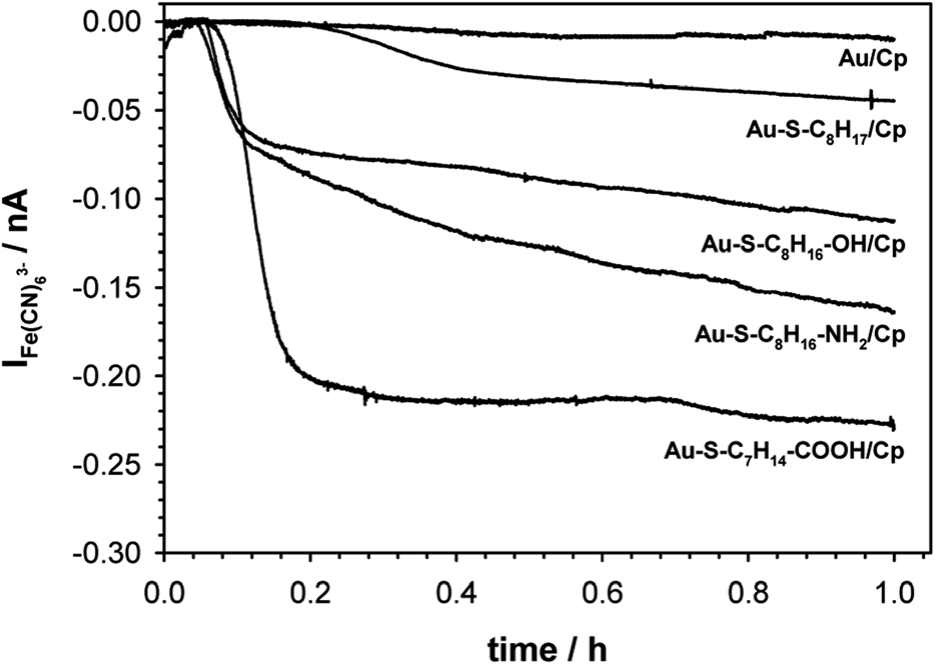
Chronoammperograms of Fe^3+^ ions produced during catalytic reaction. Experimental conditions: 0.1 M PB (pH 7.0) with addition of 0.15 M K_2_SO_4_, gold microelectrode (*ϕ* = 10 μm), *E*_appl._ = 80 mV s^−1^.

It is known that the efficiency of bioelectrocatalysis is influenced by the orientation of the enzyme *versus* the electrode surface. Hydrophilic surfaces, obtained through the presence of functional groups like: –OH, –NH_2_, –COOH, contrary to hydrophobic one, favour the reorientation and rearrangement of adsorbed enzyme molecules and in consequence should modulate its catalytic activity.^56^ The measurements concern the catalytic activity of ceruloplasmin were carried out in a two potentiostats system: the first potentiostat, by applying a 220 mV to the electrode with ceruloplasmin, was to maintain the copper active center of ceruloplasmin at the +II oxidation state, so that throughout the duration of the measurement ceruloplasmin would be catalytically active, and oxidize iron(ii) ions present in the solution; second potentiostat was used to monitor the currents generated during the catalytic reduction of iron(iii) ions by ceruloplasmin, according to the diagram shown in Scheme 2. The recorded iron(iii) reduction current was a measure of the electrocatalytic activity of ceruloplasmin. For this purpose, chronoamperometric curves were recorded for ceruloplasmin immobilized on individual thiol layers. Based on the recorded chronoammperograms, shown in Fig. 6, it can be seen that with the increase in hydrophilicity of the applied thiol layers to which ceruloplasmin was attached, the values of iron(iii) reduction currents increased. The highest value of the iron(iii) reduction current was recorded when ceruloplasmin was immobilized on an electrode modified with thiol layer with –COOH terminal group. On the other hand, the lowest values of the iron(iii) ions reduction current were recorded for ceruloplasmin immobilized on the 1-octanothiol layer. The direct adsorption of Cp on the bare gold surface caused inhibition of the enzyme activity of the enzyme *versus* [Fe(CN)_6_]^4−^ species. From the literature it is known that the contact of the enzyme with metal surface can leads to the various behaviour of the enzyme: deactivation, or the absence of heterogeneous electron transfer, or the formation of an inactive form of the enzyme.^57–59^ Moreover, the rate of deactivation of “blue” multicopper oxidases strongly depends on the crystallographic structure of gold. The Pankratov and co-workers proved that this process is the fastest at Au(111) and the slowest at polycrystalline.^60^

The observed trend in the changes of the value of Fe(iii) reduction current proved that only the highly hydrophilic layers allow to reach the most favourable orientation of the enzyme for catalytic process. The influence of the substrate surface hydrophilicity on the enzyme orientation was confirmed by PMIRRAS studies.

Ceruloplasmin belongs to the group of “blue” multicopper oxidases like *e.g.* laccase, bilirubin oxidase or ascorbate oxidase.^61^ The catalytic activity of such class of enzymes strongly depends on the orientation of the enzyme molecule on the electrode surface. Moreover, the copper ions included in the prosthetic group are responsible not only for the electroactivity of ceruloplasmin and its catalytic properties, but also for the paramagnetic properties of this enzyme. The T1 and T2 copper centers have paramagnetic properties, while the T3 copper centers are diamagnetic.^62,63^ Thus, the orientation of the enzyme can be influenced by controlling the hydrophilicity of the substrate or by introducing a magnetic surface modifier coupled to an external magnetic field.^64,65^ The literature describes systems with the use of modifiers of different hydrophilicity, mainly functional carbon nanotubes, graphene, and phenyl layers.^66–68^ The vast majority of these studies concern laccase. However, the conclusions drawn from these studies are identical to those presented in this paper; the hydrophilic surface enhances the enzyme activity.

## 4. Conclusion

Our research perfectly match the problem of effective immobilization of the enzymes (in the most effective, from the catalytic point of view, orientation) is present in many fields of science especially in biotechnological processes with the use of effective catalysts. The aim of the research was to check the influence of the hydrophilicity of the layer modifying the electrode surface on the activity of the physically immobilized enzyme on the example of the ceruloplasmin. Taking into account the domain structure of ceruloplasmin and the fact that particular domains due to the presence of various amino acids differ in hydrophilic/hydrophobic and/or charge, the changes in substrate surface properties has influence on the orientation of the enzyme molecule on the substrate. In consequence the active site of ceruloplasmin immobilized on thiol layers of different hydrophilicity was more or less accessible to the ongoing redox reaction, as a result of which ceruloplasmin showed differentiated catalytic activity. The obtained results proved that ceruloplasmin showed the highest electrocatalytic activity when it was immobilized on the thiol layer with carboxyl terminal group, and the lowest on the thiol layer without functional groups. Such behaviour may result from a change in orientation or structural changes of ceruloplasmin occurring during the adsorption process on substrates characterized by different hydrophilicity. The QCM-D and PMIRRAS measurements confirmed that the observed changes in catalytic activity of ceruloplasmin molecules immobilized on substrates of different hydrophilicity were related to the change in orientation not the amount of adsorbed ceruloplasmin. We proved that even without formation of covalent bonds we are able to control the orientation of the immobilized enzyme.

## Author contributions

Conceptualization, A. K. and A. M. N.; methodology, A. K. and A. M. N.; formal analysis, A. K. and A. M. N.; investigation, A. K. and A. M. N.; resources, A. K.; writing – original draft preparation, A. K., C. Y. and A. M. N.; writing – review & editing, A. K., C. Y. and A. M. N.; visualization, A. K. and A. M. N.; funding acquisition, A. K.

## Conflicts of interest

There are no conflicts to declare.

## Supplementary Material
